# Understanding the Roles of over 65-Year-Old Male and Female Carers: A Comparative Analysis of Informal Caregiving

**DOI:** 10.3390/geriatrics10030075

**Published:** 2025-05-29

**Authors:** Purificación Ballester, Clara Pérez-Esteve, Alicia Sánchez-García, Eva Gil-Hernández, Mercedes Guilabert, José Joaquín Mira

**Affiliations:** 1 Faculty of Pharmacy and Nutrition, Department of Clinical Pharmacology, Universidad Católica de San Antonio de Murcia (UCAM), 30107 Murcia, Spain; maria.ballester04@umh.es; 2 ATENEA Research Team, Foundation for the Promotion of Health and Biomedical Research of the Valencian Region (FISABIO), 03013 Alacant, Spain; clara.pereze@umh.es (C.P.-E.); eva.gilh@umh.es (E.G.-H.); 3 Health Psychology Department, Universidad Miguel Hernández, 03202 Elche, Spain; alicia.sanchezg@umh.es (A.S.-G.); mguilabert@umh.es (M.G.)

**Keywords:** older adults, care home, carers, gender, patient safety

## Abstract

**Background/Objectives**: This study aims to explore how gender influences the role of informal caregivers aged 65 years, considering the increasing involvement of men in caregiving due to longer life expectancy and societal norms. **Methods**: A two-year cross-sectional study was conducted in the Valencian Community, Spain, involving informal caregivers of 65 years of age and older who provided home-based care for dependent individuals with chronic conditions. The participants were recruited through public health schools, carers’ associations, and clinical consultations. The caregivers completed a comprehensive semi-structured interview, which included items from the Zarit Brief Scale (seven items) to assess caregiver burden and questions about their caregiving responsibilities, training, and experience, as well as the self-perceived frequency of medication errors. **Results**: A sample of 80 caregivers over 65 years old was analyzed, including 23 men (28.8%) and 57 women (71.2%). Male caregivers were significantly less experienced (mean = 3.1 years, SD = 5.9) compared to female caregivers (mean = 10.1 years, SD = 13.0; *p* = 0.004). Men reported lower emotional and physical burdens than women (*p*-value = 0.003), as reflected in the Zarit scores. Caregiving performance, measured by self-reported errors, was comparable between genders. **Conclusions**: This study explores the growing role of older male caregivers, highlighting their lower experience and training compared to those of women but similar caregiving performance and lower burden. Additionally, trained caregivers demonstrated significantly lower odds of experiencing burden, underscoring the importance of training as a modifiable factor. The findings emphasize the need for gender-sensitive support and tailored training programs to address disparities, reduce caregiver burden, and enhance caregiving quality and equity.

## 1. Introduction

In recent decades, our societies, in order to face the increment in life expectancy, have undergone significant transformations, due in part to a new perspective on gender roles and the progressive aging of the population. These challenges have rarely been analyzed together, though data suggest that their interaction warrants greater attention than it has received so far.

First, the number of dependent individuals requiring home care from informal caregivers within their support network is increasing significantly each year. In addition, particularly after the pandemic, more older people are choosing to age at home [1]. Second, the traditional life expectancy gap between men and women has been narrowing in Europe [2], meaning that more men are now taking on the role of informal caregivers at home. Third, despite that, it is necessary to confront directly the persistent notion that masculinity is incompatible with household tasks, a sentiment that has shaped the life course of many men and limited their involvement in caregiving tasks [3], as this role is often perceived as inherently feminine [4,5]. Fourth, evidence indicates that men and women do not experience the same level of stress as informal caregivers [6,7], which could affect the quality of care received by the care recipient. Novelty, imbalance in tasks, and duty proportion impact on the quality and efficacy of the labor delivered [8]. Fifth, we are expanding our understanding of the unintentional errors that informal caregivers make in managing medication or providing home care [9,10]. A key contributor to these errors is the lack of adequate training, which not only affects the well-being of the care recipients but also undermines caregivers’ confidence and self-perception in their role.

Subjected to the former gender bias, women have played a central role in home caregiving, while men have been less involved in these tasks [6,11]. Women have tended to take responsibility for domestic tasks and the health care of the family, including both children and husbands, even when both partners were engaged in paid work [12]. However, it is now increasingly common for men to assume the role of primary caregivers for their partners at home when they present a higher level of incapacity and dependency. This shift in domestic responsibilities presents new challenges for deinstitutionalization policies, as it may increase risks for dependent women and undermine equity if older men prematurely give up their role as informal caregivers due to their unfamiliarity with many necessary household and caregiving tasks [8].

### 1.1. Older People Care by Family Caregivers in Spain

Over 20% of Spain’s population is over 65, a figure expected to grow significantly in the coming decades. Spain’s strong familial culture emphasizes family responsibility for elder care, with many older adults preferring to remain at home, supported by relatives [13]. Recipients with limited autonomy may receive government financial assistance to cover the costs of hiring an informal caregiver at home or to provide economic compensation to a family member who takes on this role. In recent years, a growing number of immigrant women, often without formal caregiver qualifications, have joined the ranks of informal caregivers. This model faces challenges from increasing care demands, evolving family structures, economic pressures, and limited access to professional services.

### 1.2. Gender Gaps in Informal Caregiving

Understanding these dynamics and their consequences is crucial for developing policies and support programs for older people, particularly considering the evolving nature of the care economy. A better understanding of the challenges faced by caregivers over 65 years old, considering the socioeconomic changes in developed countries, would allow for more effective policy recommendations that could improve the well-being of both care recipients and their caregivers. Programs designed to train informal caregivers should account for these differences and provide tailored support to meet the needs of diverse caregiver profiles. This study explores gender differences in informal caregiving roles among older adults, focusing on the proportion of men and women involved, their caregiving motivation, training received, emotional burden, time for self-care, and caregiving performance, including self-reported errors. This study aims to provide a comprehensive understanding of gender disparities and identify opportunities for targeted support and training for caregivers over 65. In this context, it explores how gender influences the role of informal caregivers in this age group, considering both the traditional expectation that women may be more prepared for caregiving due to social norms and life experiences and the increasing involvement of men as a result of longer life expectancy and evolving social roles.

## 2. Materials and Methods

This cross-sectional study employed a semi-structured interview conducted between June 2023 and September 2024, completed by informal caregivers over 65 years old with at least one year of experience, caring for dependent older patients with chronic conditions. The study was conducted in the Valencian Community, Spain, and included caregivers living in urban areas with populations ranging from 10,000 to 300,000 inhabitants.

### 2.1. Definitions

In this study, a medication error was defined as an event related to the use of medication at home by an informal caregiver that involved violations of treatment prescriptions (e.g., incorrect medication dosage, timing, or way of administration) and had the potential to result in adverse health outcomes for the recipient, such as medication inefficacy, side effects, or serious complications. These incidents may require adjustments in the ongoing medication, close recipient monitoring, or in more severe cases, medical intervention or hospitalization or may produce lasting adverse health effects in the patient.

An informal caregiver is described by BetterCare, a European Network focused on enhancing safety caregiving at home, funded by COST (European Cooperation in Science and Technology, Brussels, Belgium), as an individual who provides care at home to patients with chronic illnesses, disabilities, or other long-term health or care needs, without having formal qualifications or academic training as a healthcare professional. These caregivers can be family members or close friends offering support within the context of a personal relationship but can also include individuals who are not personally related to the care recipients. This kind of care can be either paid or unpaid. Qualified caregivers are those who have received a specific qualification or training for 20 h or more, whose content is centered on strategies or knowledge related to caring for patients.

Informal caregiver burden refers to the stress and strain experienced by individuals providing care to a dependent person, often leading to emotional exhaustion, role strain, and a reduced quality of life [14].

The level of intrinsic motivation in caregiving tasks is defined as the caregiver’s perceived ability to effectively manage medication and care responsibilities.

### 2.2. Participants

In this study, the informal caregivers (over 65 years old) were individuals providing home-based care, including unpaid caregivers (primarily relatives of the recipient or personnel from non-profit associations) and paid caregivers, who were typically relatives or untrained personnel (e.g., immigrants) who were compensated for their caregiving work through the official subsidies received by the care recipients.

The participants were recruited through two main approaches. First, caregiver associations, chronic patient associations, home care service, and public health schools that offer courses and training programs for informal caregivers sent invitation messages to their members or participants. Second, recruitment took place in primary care consultations, where chronic patients accompanied by an informal caregiver were informed about the study and given the option to participate. Inclusion criteria: informal caregivers actively providing care and administering medications to a person for at least the past year. Exclusion criteria: caregivers who did not speak Spanish and those trained as health and care workers and who provided professional medical care at home, such as visiting nurses, physiotherapists, social workers, psychologists, or other institutional workers performing medical procedures in home settings.

The sample size of 80 participants was calculated using a standard formula to determine the minimum number of participants needed to identify significant differences between the proportions of male and female informal caregivers. It was calculated considering an expected proportion of men of 30%, with a 95% confidence level and a margin of error of 10%. Semi-structured, in-person interviews were conducted until the required sample size was reached, with recruitment carried out using the snowball sampling technique. No incentives were provided to the participants for their involvement.

### 2.3. Materials

The interview content was developed based on the researchers’ team previous experience [15,16], a thorough review of pertinent literature, and online consultations with three experts in the fields of health psychology, pharmacy, and public health. The survey comprised 51 fixed, 5 multiple-choice, and 3 open-ended questions. The fixed and multiple-choice questions gathered information on caregiver gender, dedication, training, years of experience, use of external aids for medication management, and the level of dependency assigned to the patient, along with whether the patient was entitled to official subsidies to cover the caregiving expenses, among other details. The open-ended questions allowed caregivers to report any issues related to medication, care, or aids during the interview.

The characterization of medication errors and their number, acknowledged by the caregivers in the last six months, was evaluated as the performance level. The degree of recipient dependence, the caregiver’s motivation to provide care, and the emotional burden associated with caregiving were considered factors influencing the interpretation of the results. Additionally, the study took into account whether the caregiver received any governmental support for their caregiving role. The variables included in the study and their operational definitions are provided in Table 1.

The level of dependency assigned through an assessment by technicians from the Spanish public social protection system was provided by the caregiver themselves. Furthermore, the Barthel scale was used to determine the recipient’s level of dependency [17] in performing basic activities of daily living, assigning a score based on the level of assistance required, ranging from total independence to complete dependence. Also, the interview explored caregivers’ skills and knowledge [18], relevant concepts regarding medication and care, such as their perception of a spontaneous adverse event inception [19], or medication prescriptions accomplishment [20]. The degree of intrinsic motivation in caregiving tasks was assessed using a single-item scale ranging from 0 to 10, where 0 indicated “not motivated at all”, and 10 indicated “completely motivated”. This aspect was included considering its potential influence on caregiver burden and medication management performance [21]. Additionally, it assessed the caregivers’ emotional status and burden [22], using the Zarit scale [23]. The original 22-item Zarit version has a high internal consistency (Cronbach’s alpha ≥ 0.80) and has been adapted into several shorter versions, including a 12-item (ZBI-12) and a 7-item (ZBI-7) versions, while maintaining good reliability and validity [24,25]. These versions have been validated in various languages and cultural contexts, including the Spanish ones. In this study, to minimize respondent fatigue, we opted for the ZBI-7 version, whose items also better aligned with the objectives of our research. Each item of the 7-item (ZBI-7) version was rated on a 5-point Likert scale ranging from 0 (never) to 4 (almost always), resulting in total scores between 0 and 28. The scores on the Zarit scale were considered to split the sample according to the self-reported burden and associate the latter to gender. Caregivers were considered to have a burden when they scored higher than 14 on the shortened Zarit scale. This cutoff point was established considering the cutoff criteria of the widely used 22-item Zarit version, where a score of 47 indicates moderate to severe burden. Based on this reference, we calculated a proportional cutoff for the 7-item version, classifying caregivers with scores above 14 as experiencing some degree of burden (moderate or severe).

To collect the responses, an online platform was developed, allowing the participants to access it freely and anonymously at any time after receiving an invitation. The platform was accessible from any internet-connected device and included safeguards to prevent multiple responses from the same device IP address. However, no cookies were used.

### 2.4. Procedure

The participants were invited to take part in the study through an official invitation letter, ensuring confidentiality and anonymity. Those who agreed attended an informative session where they received details about the study and the participation process. The participants could choose between two response methods. A small number opted to complete the questionnaire independently from home using an online platform accessed through a provided link. However, the majority preferred assistance in completing the questionnaire. In this case, three trained psychologists guided them through the process using a paper-and-pencil format. Once completed, the psychologists manually transferred the responses to the digital database on behalf of the participants.

Assisted interviews were conducted within the premises of the participating institutions under conditions of confidentiality. The psychologists, who were experienced in conducting semi-structured interviews, were not affiliated with the associations, institutions, and centers where the participants received healthcare or social assistance. Both those who chose to respond independently and those who requested assistance answered the same set of questions, ensuring consistency across all responses. This approach ensured data accuracy, minimized potential response biases, and provided support to the participants who preferred assistance in answering the survey.

### 2.5. Statistical Analysis

First, missing data within the dataset were addressed by excluding incomplete records. Caregivers who did not respond to at least 80% of the interview questions were excluded. Participants’ wishes to withhold their demographic data to ensure anonymity were respected. After data curation, quantitative results were expressed using descriptive statistics. Descriptive analyses were conducted both globally and stratified by gender to compare outcomes between men and women. Absolute and relative frequencies were calculated for each relevant variable. Caregiver burden was assessed using the ZBI-7 score, both as a continuous variable and as a categorical variable. For categorical analysis, the participants were classified into two groups based on whether they experienced a burden (ZBI-7 score > 14) or not. Caregivers were considered inexperienced if they had less than two years of caregiving experience, while those with two or more years were classified as experienced.

Bivariate statistics were employed to explore relationships between gender and other key variables, including overall burden, motivation, and caregiver training levels and experience. Fisher’s exact test was applied for categorical variables when subgroups had very small sample sizes, while the chi-square test was used for larger categorical comparisons. The Mann–Whitney U test was employed for non-parametric continuous variables, as these did not follow a normal distribution. To evaluate the association between gender and error frequency, relative risk (RR) and absolute risk difference (excess risk) were calculated as measures of association, comparing male and female caregivers within each experience group. A logistic regression analysis was performed to explore the factors influencing caregiver burden, using variables such as education, experience, gender, relationship to the care recipient, and others as independent variables. A separate logistic regression analysis was conducted using the same method to identify factors associated with medication errors, aiming to determine variables linked to better performance and reduced likelihood of errors. For the logistic regression models, predictor variables were selected based on their theoretical relevance and potential influence on the dependent variables, as identified in previous literature and caregiving research. Additionally, variables were included if they demonstrated significant associations in preliminary analyses or were considered key factors in understanding caregiving burden and medication management. Interaction terms were incorporated when prior theoretical rationale suggested that the effect of one predictor might depend on the level of another (e.g., motivation × training). This approach allowed us to explore potential associations and assess the relative influence of each predictor on the outcome, while ensuring that the model included clinically and contextually meaningful variables. Statistical significance was defined as a *p*-value of less than 0.05.

## 3. Results

Eighty-two participants responded to the survey. Two subjects were excluded due to missing data in more than 80% of the fields, which resulted in a final sample of 80 carers. Among them, 23 (28.8%) were male, with a mean age of 69.7 years (SD 5.6; Table 2).

### 3.1. Relationship with the Recipient of Care

In this sample, fewer men (8.7%) than women (17.5%) were hired as caregivers, although the difference was not statistically significant (*p*-value = 0.259). For most male caregivers, caregiving was their main activity (69.6%), similar to what observed for female caregivers (Table 2). A higher proportion of men (87.0%) than of women (77.2%) lived with the care recipient, but this difference was also not statistically significant (*p*-value = 0.376). Additionally, fewer men (8.7%) than women (17.5%) received payment for their caregiving work, with no significant difference observed (*p*-value = 0.259).

### 3.2. Training Received to Be a Caregiver

Male caregivers had received similar levels of training as female caregivers (*p*-value = 0.505). However, more male caregivers (87.0%) had no training compared to the female caregivers (75.4%; Table 2). Male caregivers also had significantly fewer years of experience than their female counterparts (3.1 years, SD = 5.9 vs. 10.1 years, SD = 13.0; *p*-value = 0.004), indicating that men typically begin caregiving later in life (Table 1).

### 3.3. Motivation to Assume the Role of Informal Caregiver at Home

When assessing the motivation to take on the role of informal caregiver at home, men appeared more motivated than women, with scores of 5.5 (SD = 4.1) and 4.4 (SD = 3.7) out of 10, respectively (Table 2). However, this difference was not statistically significant (*p*-value = 0.239).

### 3.4. Caregiver’s Overall Burden

Regarding the caregiver’s overall burden associated with caregiving responsibilities, we can state that females experienced significantly higher levels of burden compared to males, as reflected in their Zarit scores (Table 3). Female caregivers reported feeling significantly more overwhelmed than men when trying to balance their caregiving responsibilities with other commitments, such as work and family (*p*-value = 0.003). They also felt that their health had worsened due to the caregiving responsibilities more than men (*p*-value = 0.001) and experienced greater tension when being with the person they cared for compared to men (*p*-value = 0.028). Additionally, female caregivers reported feeling that they had lost control of their lives since taking on the caregiving role more than men (*p*-value = 0.006) and perceived their caregiving role as more of a “burden” compared to male caregivers (*p*-value = 0.003). Additionally, 39.2% of male caregivers reported not feeling fatigued with their work, compared to 7.0% of female caregivers (*p*-value = 0.003) (Table 3). This was further supported by the fact that 44 (77.2%) female caregivers experienced burden due to their caregiving work (ZBI-7 score > 14 points), compared to 11 (47.8%) male caregivers.

### 3.5. Factors Influencing Caregiver Burden

Table 4 presents the results of a logistic regression analysis examining the factors associated with the likelihood of experiencing caregiver burden. Being female was significantly associated with an increased probability of burden, with women having more than six times the odds of experiencing caregiver burden compared to men (OR = 6.257, *p* = 0.006). Furthermore, caregivers who had received training showed substantially lower odds of reporting burden (OR = 0.196, *p* = 0.005), suggesting a protective effect of caregiver education. Motivation to provide care also emerged as a significant protective factor, with higher motivation associated with lower odds of experiencing burden (OR = 0.638, *p* = 0.009). Notably, a significant interaction was found between training and motivation (OR = 1.243, *p* = 0.039), indicating that the beneficial effect of training was moderated by the caregiver’s level of motivation. Specifically, the protective effect of training was strongest among caregivers with lower motivation and diminished as motivation increased. Other variables, such as salary, daily caregiving hours, and living with the care recipient, did not show statistically significant associations with caregiver burden in this model.

### 3.6. Impact of Motivation on Error Likehood

The factors influencing the probability of errors were analyzed using logistic regression, revealing that motivation was the only variable with a significant impact on the likelihood of errors in caregiving (Table 5).

The results show that an increase in motivation significantly reduced the probability of making an error. The motivation coefficient was −0.179 (*p*-value = 0.021), indicating that for each additional point in motivation, the probability of making an error decreased by 19.6% (odds ratio = 1.196, 95% CI 1.033–1.406). These findings suggest that higher motivation is associated with a lower risk of making errors, which could have important implications for improving performance and reducing errors among informal caregivers.

### 3.7. Level of Performance

Nine male caregivers (39.1%) and nineteen female caregivers (33.3%) acknowledged making a medication or caregiving error in the past six months (Table 6). The rates of acknowledged errors committed in the last 6 months among male and female caregivers did not significantly differ (*p*-value = 0.816).

When considering caregivers with less experience, men showed a 16.2% higher absolute risk of error compared to women without experience (Table 6). However, the 95% confidence interval for this difference included the null value, indicating that the difference was not statistically significant.

### 3.8. Type of Reported Errors

A total of 54 errors were reported, with 24.1% self-attributed by male caregivers and 75.9% by female caregivers (Table 7). The most frequently reported medication error was omission (13.8%), followed by timing errors (12.5%). Under-dosing occurred in 6.3% of the cases, while confusion (5.0%) and over-dosing (2.5%) were less common. The differences between male and female caregivers in medication errors were not statistically significant. In turn, the most common caregiving error was back injuries sustained during improper transfers or movements of the care recipient (11.3%). Other reported errors included forgetting dietary requirements (7.5%), allowing for falls during bathing (6.3%), and failing to achieve the appropriate food texture (2.5%). Gender differences in caregiving errors were also not statistically significant.

## 4. Discussion

The profile of older male caregivers assuming home caregiving roles has received limited attention in the literature, primarily due to the traditional association of caregiving with women, shaped by entrenched societal and cultural gender biases [8]. This study examined the demographic characteristics, caregiving roles, and the emotional and physical burdens of male and female caregivers 65 years old and over.

While the initial assumption that inexperienced men over 65 may be less equipped to handle the demands of caregiving compared to women in the same age group is not definitively supported due to the small sample size, the observed data suggest a possible trend in this direction. This trend is further supported by the lack of significant differences in performance among experienced caregivers, indicating that with time and practice, gender-related disparities in caregiving may decrease. This suggests that the observation made in this study is worth being explored in more detail, despite the challenges of enrolling male caregivers who provide care for their partners.

While both groups share common challenges, the results suggest gender differences in several aspects of caregiving, particularly in experience level, emotional burden, and self-care time. The findings indicate that male caregivers often begin caregiving later in life and report a lower emotional burden. In contrast, female caregivers appear to face greater physical and emotional challenges, which may highlight the need for targeted interventions. Despite these differences, caregiving performance, measured by errors, appeared comparable across genders.

### 4.1. Gender Differences in Caregiving Experience and Emotional Burden

The findings of this study indicate that men over the age of 65 who take on the role of informal caregivers often have less experience with domestic caregiving tasks compared to women in similar roles, as previous reported [1,8]. This aligns with recent publications that describe how men typically begin caregiving later in life, usually for a close relative and often without receiving any income or financial compensation [26]. This late entry into caregiving reflects societal norms that traditionally exclude men from domestic responsibilities. In contrast, women often assume caregiving roles earlier in life, which leads to cumulative emotional and physical demands [4,27,28]. Our results indicate that being female is associated with approximately a fivefold increase in the odds of experiencing caregiver burden compared to being male. This trend may reflect the greater caregiving demands typically placed on women, possibly influenced by societal expectations regarding caregiving as a traditionally female role [8]. These findings align with the existing literature on caregiver burden [26,27,28,29].

In this study, the Zarit scale results further emphasize these disparities, with women reporting significantly higher levels of emotional exhaustion, fatigue, and perceived loss of control over their lives. These findings align with previous studies showing that women are more prone to depressive symptoms, sleep disruption, and feelings of being overwhelmed due to caregiving responsibilities [11,29]. Female caregivers with male recipients reported even higher levels of distress, exacerbated by societal pressures and caregiving expectations [4,8]. These outcomes suggest that female caregivers may experience a dual strain: first, from the caregiving tasks themselves, and second, from societal expectations related to the caregiving roles. Interestingly, male caregivers reported lower emotional distress, which may be attributable to differing coping strategies or societal expectations that prioritize their self-care [11,29,30]. Nevertheless, it should also be considered that this trend has been recently reported in the literature, where Schaffler-Schaden and colleagues have linked the burden scale (BSFC-s) with the number of years dedicated to caregiving [31].

### 4.2. Caregiving Performance—Motivation and Training as Protective Factors

Despite these gender differences, caregiving performance, measured by self-reported errors, did not differ significantly between men and women. This suggests that women, despite their heavier emotional burden, maintain similar levels of performance to those of men. Technology, in the future of home care, could help women to alleviate their emotional burden, given the multidimensionality and complexity of home care as well as the challenges and strains involved [32]. The data in the logistic regression model suggested that motivation and/or formal training may play a protective role in reducing caregiver burden. The data show that higher motivation was associated with a lower risk of making errors, suggesting that motivated caregivers are better equipped to manage the complexities of caregiving; these results are aligned with previous published work [33]. Additionally, the duration of caregiving plays a key role, as longer caregiving periods are often associated with increased strain but can also reinforce coping strategies and caregiving skills over time [34]. Expectations regarding the caregiving roles serve as a key mediator between the demands of caregiving and the outcomes, such as informal caregiver burnout [29,35]. Formal training also emerged as a significant factor in alleviating caregiver burden. Caregivers who completed more than 20 h of training were significantly less likely to report experiencing burden. Training may provide caregivers with essential skills and knowledge, potentially increasing their confidence and reducing the perceived challenges of caregiving. This observation aligns with previous research, highlighting the importance of structured training program [33].

While male caregivers generally have less experience and are less likely to seek formal training, these programs could be particularly beneficial for them, helping to bridge gaps in their preparedness and ensuring better caregiving practices. For female caregivers, who often face greater cumulative emotional and physical demands, training could serve as a critical buffer, providing strategies to manage stress and caregiving tasks more effectively. It is important to consider that, compared to previous studies, younger male caregivers are more likely to admit to a medication error within the past six months [36]. These findings highlight the necessity of accessible and structured training initiatives to support caregivers and improve caregiving outcomes in both genders.

### 4.3. Familial Roles and Compensation

This study also found that older caregivers, regardless of their gender, were primarily relatives of the care recipient, with men most commonly caring for their spouses [28]. This reflects the strong familial nature of caregiving for older male adult carers. This hypothesis is further supported by the fact that fewer male carers reported receiving financial compensation compared to women, highlighting potential inequities in how caregiving is valued within families and society [4,28]. This disparity suggests the need for policies that recognize and support informal caregiving roles, regardless of gender, to reduce the financial and emotional strain.

### 4.4. Practical Implications

It is important to be vigilant when an older man assumes caregiving responsibilities, as he may be unfamiliar with basic caregiving standards or practices. There is also a higher likelihood that he will not seek training or education, potentially due to perceiving it as inappropriate or viewing caregiving as a “women’s task”, which can act as a barrier that must be considered. Support strategies to address the emotional burden should be differentiated, as women in caregiving roles often require greater and sooner attention in this regard than men. The results from this study suggest that caregiver training may help reduce this burden. Expanding accessible training programs with a focus on practical skills, stress management, and caregiving confidence could be beneficial for improving caregiver well-being. Future research should confirm the challenges faced by and the training needs of caregivers according to gender, and then it will be worth exploring gender-specific training. This could be tailored to address the potential challenges faced by female caregivers and bridge equity gaps. Healthcare professionals must address the risks of errors in home care, including medication management, by providing clear instructions to informal caregivers. Additionally, promoting shared caregiving responsibilities within families could help distribute the burden more equitably, reducing the disproportionate demands placed on women.

### 4.5. Limitations

This study has several limitations. First, the snowball sampling method is a non-probability technique, meaning that the sample was not randomly selected and is unlikely to be representative of the broader population of informal caregivers. As a result, the findings should be interpreted with caution, as they have limited generalizability. Second, the cross-sectional design restricts our ability to draw causal inferences about the observed associations between caregiving factors and outcomes. Third, some key variables were measured using single-item questions rather than validated multi-item scales. This may limit the reliability and validity of the measures and warrants careful interpretation. Fourth, information related to education, social beliefs, or similar contextual factors was not collected, in full respect of the participants’ privacy and in accordance with the principles of voluntary and anonymous participation. Additionally, the sampling method may have introduced an unintentional selection bias, potentially overrepresenting certain caregiver profiles (such as middle-aged women in family settings) while underrepresenting others, such as older male caregivers. Finally, this study should be considered an initial exploration, constrained by the challenges of reaching underrepresented caregiving populations.

### 4.6. Future Directions

Although extensive research exists on caregiving, significant gaps remain in understanding why some individuals take on caregiving roles more actively than others, how they navigate these responsibilities, and the long-term consequences of caregiving. The safety of the care recipients under the care of unqualified and untrained informal caregivers has received little attention, despite its significant impact on the well-being of both the recipient and the caregiver, as well as on healthcare resource utilization. Gaining insight into these dynamics is essential for designing effective policies and interventions that address the multifaceted challenges of caregiving, particularly for women, who often endure compounded emotional and physical strains [37,38].

Additionally, the preliminary trends observed in this study, suggesting possible gender differences in the capacity to adapt to the caregiving roles, underscore the need for further research. Given the difficulty in enrolling male caregivers who provide care to their partners, targeted efforts to better understand their experiences are needed. This could inform the development of gender-sensitive support programs aimed at reducing disparities in caregiving outcomes and improving the overall quality of informal care.

Future research should prioritize exploring the longitudinal effects of caregiving, with a specific focus on the impact of training and support systems. Additionally, investigating how societal and cultural norms shape the caregiving dynamics can provide a valuable context for addressing inequities and improving caregiver outcomes. Expanding this research to include more diverse and larger samples would offer a broader and more nuanced understanding of caregiving challenges and opportunities, ultimately guiding the development of targeted and inclusive interventions.

## 5. Conclusions

In this study, significant gender differences in older informal caregiving were identified, particularly in experience levels, emotional burden, and time for self-care. Male caregivers over 65 years old tend to start caregiving later in life and reported lower emotional burden, whereas female caregivers appeared to face substantial physical and emotional challenges, including a fivefold greater likelihood of experiencing caregiver burden. Despite these differences, caregiving performance, measured by self-reported errors, remained comparable across genders.

These findings underscore the critical role of formal training as a modifiable factor to reduce caregiver burden. Trained caregivers demonstrated significantly lower odds of experiencing burden, highlighting the need to prioritize the implementation of comprehensive training programs. Such programs could be particularly beneficial for male caregivers, bridging gaps in experience and preparedness, while also serving as a buffer for female caregivers, who often face cumulative emotional and physical strain.

Future efforts should focus on developing gender-sensitive support systems that address the unique challenges faced by male and female caregivers. Tailored interventions, such as psychological counseling, respite care, and skill-based training programs, could alleviate the emotional burden on women while improving caregiving confidence and competence among men.

By addressing these disparities, it will be possible to foster equity in caregiving roles and enhance the quality of care for dependent individuals. These efforts are essential to adapt to the shifting societal expectations and demographic trends, including increasing male participation in caregiving roles as life expectancy continues to rise.

## Figures and Tables

**Table 1:** Variable definitions.

Variable	Definition
**Informal caregiver’s overall burden**	Overall burden experienced by an informal caregiver due to caregiving responsibilities, measured using the 7-item Zarit Burden Scale (ZBI-7).
**Motivation**	Self-perceived motivation to achieve good caregiving outcomes, measured through agreement with the statement “Do you feel motivated for what you do to have a good result?” using a Likert-type scale.
**Caregiving as the main activity**	Whether caregiving is the caregiver’s main daily occupation, recorded as yes or no.
**Salary**	Indicates whether the caregiver is financially compensated for caregiving, categorized as yes, no, or prefers not to answer.
**Training as a caregiver**	Formal training received related to caregiving tasks, categorized as none or yes (1–20 h).
**Recipient’s place of residence**	Geographic classification based on population size, categorized as large city (>100,000), medium city (10,000–100,000), or small city (<10,000).
**Caregiver’s use of a pillbox**	Whether a pillbox is used to manage medications, recorded as yes or no.
**Recipient government assistance**	Whether the recipient receives official assistance after formal assessment, recorded as yes or no.
**Error awareness**	Whether the caregiver reports having made caregiving- or medication-related mistakes in the last six months, recorded as yes or no.
**Medication errors**	Specific mistakes made in administering medications, categorized with multiple response options: under-dosing, over-dosing, omission, timing, confusion.
**Caregiving errors**	Specific caregiving-related mistakes committed by the caregiver, categorized with multiple response options: dietary error, food texture error, bathing fall, or transfer injury.

**Table 2:** Sample.

	Total	Male Carers	Female Carers	
	*N* (%)	*N* (%)	*N* (%)	*p*-Value *
Gender of the carer	80 (100)	23 (28.8)	57 (71.2)	<0.001
Age of the carer (M, SD)	69.2 (SD 5.9)	69.7 (SD 5.6)	69.0 (SD 6.1)	0.659
Caregiving as the main activity				
Yes	57 (71.3)	16 (69.6)	41 (71.9)	1.000
No	23 (28.8)	7 (30.4)	16 (28.1)	
Training as a caregiver				
None	63 (78.8)	20 (87.0)	43 (75.4)	0.368
1–20 h	17 (21.3)	3 (13.0)	14 (24.6)	
Relative of the recipient				
Yes	73 (91.3)	22 (95.7)	51 (89.5)	0.667
No	7 (8.8)	1 (4.3)	6 (10.5)	
Carer relationship to the recipient				
Spouse	57 (78.1)	20 (90.9)	37 (72.5)	0.149
Child	9 (12.3)	2 (9.1)	7 (13.7)	
Other	7 (9.6)	0 (0.0)	7 (13.7)	
Number of recipients being cared for				
One	78 (97.5)	23 (100)	55 (96.5)	0.204
Two or more	2 (2.5)	0 (0.0)	2 (3.5)	
Salary for caregiving work				
Yes	12 (15.0)	2 (8.7)	10 (17.5)	0.259
No	67 (83.8)	20 (87.0)	47 (82.5)	
Prefers not to answer	1 (1.3)	1 (4.3)	0 (0.0)	
Main carer language				
Spanish	78 (97.5)	22 (95.7)	56 (98.2)	0.495
Other	2 (2.5)	1 (4.3)	1 (1.8)	
Gender of the recipient				
Male	46 (57.5)	2 (8.7)	44 (77.2)	<0.001
Female	34 (42.5)	21 (91.3)	13 (22.8)	
Age of the recipient (M, SD)	73.5 (SD 14.9)	71.6 (SD 13.3)	74.3 (15.6)	0.101
Recipient main diagnosis				
Parkinson’s disease	18 (22.5)	6 (26.1)	12 (21.1)	0.936
Alzheimer’s disease	9 (11.3)	2 (8.7)	7 (12.3)	
Cardiovascular disease	9 (11.3)	4 (17.4)	5 (8.8)	
Cancer	7 (8.8)	1 (4.3)	6 (10.5)	
Diabetes	6 (7.5)	2 (8.7)	4 (7.0)	
Heart failure	4 (5.0)	1 (4.3)	3 (5.3)	
Reduced mobility	3 (3.8)	1 (4.3)	2 (3.5)	
Other	24 (30.0)	6 (26.1)	18 (31.6)	
Recipient’s place of residence				
Big city (>100,000 inhab.)	47 (58.8)	15 (65.2)	32 (56.1)	0.329
Medium city (10,000–100,000 inhab.)	26 (32.5)	5 (21.7)	21 (36.8)	
Small city (<10,000 inhab.)	7 (8.8)	3 (13.0)	4 (7.0)	
Recipients’ ongoing medication per day (M, SD)	8.5 (4.3)	8.1 (3.8)	8.7 (4.5)	0.906
Caregivers’ use of a pillbox				
Yes	40 (50.0)	7 (30.4)	33 (57.9)	0.048
No	40 (50.0)	16 (69.6)	24 (42.1)	
The recipient is receiving government assistance following a formal technical assessment				
Yes	40 (50.0)	11 (47.8)	29 (50.9)	1.000
No	40 (50.0)	12 (52.2)	28 (49.1)	
Residence with care recipient				
Yes	64 (80.0)	20 (87.0)	44 (77.2)	0.376
No	16 (20.0)	3 (13.0)	13 (22.8)	
Years of experience as a caregiver (M, SD)	8.1 (SD 11.8)	3.1 (SD 5.9)	10.1 (SD 13.0)	0.004
Daily caregiving hours (M, SD)	16.2 (SD 8.8)	14.5 (SD 10.1)	16.9 (8.3)	0.287
Motivation	4.8 (SD 3.8)	5.5 (SD 4.1)	4.4 (SD 3.7)	0.239

* Chi-square test, Fisher’s exact test, and Mann–Whitney U test were used appropriately according to the type of data.

**Table 3:** Informal caregiver’s overall burden measured by Zarit scale (ZBI-7).

	Total		Male Carers		Female Carers		
	Mean	SD	Mean	SD	Mean	SD	*p*-Value *
Do you feel that, due to the time you dedicate to caregiving, you do not have enough time for yourself?	3.3	1.3	2.8	1.2	3.5	1.3	0.056
Do you feel overwhelmed when trying to balance your caregiving responsibilities with other commitments (work, family)?	2.9	1.2	2.2	1.2	3.1	1.1	0.003
Do you think that caregiving has negatively affected your relationship with other family members?	2.3	1.3	2.0	1.1	2.4	1.3	0.181
Do you feel that your health has worsened due to your caregiving responsibilities?	2.6	1.3	1.8	1.0	3.0	1.2	<0.001
Do you feel tense when you are around the person you care for?	2.1	1.2	1.6	0.8	2.3	1.3	0.028
Do you feel like you have lost control of your life since you started your caregiving role?	2.5	1.3	1.9	0.9	2.8	1.3	0.006
Overall, do you feel that your caregiving role is a “burden”?	2.4	1.2	1.8	0.8	2.7	1.3	0.003
Overall score (ZBI-7)	18.4	6.6	14.1	5.0	19.8	6.8	<0.001

* Mann–Whitney U test.

**Table 4:** Factors contributing to increased burden among informal caregivers.

Factors	β Estimate	Std. Error	*p*-Value	OR	95% CI Lower	95% CI Upper
(Intercept)	4.232	1.644	0.010	68.854	3.485	2415.902
Caregiver gender	1.834	0.669	0.006	6.257	1.786	25.538
Relative of the recipient	0.558	1.034	0.589	1.747	0.256	16.926
Salary	−1.627	0.581	0.005	0.196	0.053	0.557
Training as a caregiver	−0.061	0.039	0.120	0.940	0.867	1.013
Years of experience	−0.634	1.042	0.537	0.526	0.056	3.856
Daily caregiving hours	4.232	1.644	0.010	68.854	3.485	2415.902
Residence with care recipient	1.834	0.669	0.006	6.257	1.786	25.538
Motivation	−0.449	0.171	0.009	0.638	0.439	0.870
Training and Motivation	0.217	0.105	0.039	1.243	1.039	1.601

**Table 5:** Factors contributing to an increased probability of making errors.

Factors	β Estimate	Std. Error	*p*-Value	OR	95% CI Lower	95% CI Upper
**(Intercept)**	−5.243	4.056	0.196	0.005	0.000	12.393
**Caregiver gender**	−0.043	0.642	0.946	0.958	0.273	3.474
**Caregiver age**	0.074	0.049	0.135	1.077	0.979	1.190
**Training as a caregiver**	−0.040	0.330	0.904	0.961	0.485	1.821
**Years of experience**	−0.005	0.031	0.880	0.995	0.931	1.053
**Salary**	−0.914	0.753	0.225	0.401	0.086	1.739
**Motivation**	−0.179	0.078	0.021	1.196	1.033	1.406
**Informal caregiver’s overall burden**	0.008	0.042	0.854	1.008	0.927	1.097
**Daily caregiving hours**	−0.017	0.033	0.610	0.983	0.920	1.050
**Residence with care** **recipient**	0.504	0.827	0.543	1.655	0.322	8.683

**Table 6:** Error frequency by caregiver experience and gender.

Reported Errors	Inexperienced Male	Inexperience Female	Experienced Male	Experienced Female
	*N* (%)	*N* (%)	*N* (%)	*N* (%)
No	8 (61.5)	14 (77.8)	6 (60.0)	24 (61.5)
Yes	5 (38.5)	4 (22.2)	4 (40.0)	15 (38.5)
Total	13 (100)	18 (100)	10 (100)	39 (100)
Relative Risk (RR)	1.73 (95% CI 0.57 to 5.22)		1.04 (95% CI 0.44 to 2.45)	
Excess Risk	0.162 (95% CI −0.164 to 0.489)		0.015 (95% CI −0.324 to 0.355)	

Inexperienced caregivers had been providing care for less than two years.

**Table 7:** Self-performance declared by carers and type of errors committed by caregivers.

		Total	Male Carers	Female Carers	
		*N* (%)	*N* (%)	*N* (%)	*p*-Value *
Are you aware of having made any mistakes (either in medication or in caregiving) in the past 6 months?					
	Yes	28 (35.0)	9 (39.1)	19 (33.3)	
	No	52 (65.0)	14 (60.9)	38 (66.7)	0.816
	TOTAL	80 (100)	23 (100)	57 (100)	
MEDICATION ERRORS	Under-dosing: Giving a lower dose than that prescribed to the person	5 (6.3)	2 (8.7)	3 (5.3)	0.584
	Over-dosing: Giving a higher dose than that prescribed to the person	2 (2.5)	0 (0.0)	2 (3.5)	1.000
	Omission: Forgetting to administer a dose that was prescribed to the person	11 (13.8)	3 (13.0)	8 (14.0)	1.000
	Timing: Not following the regular schedule for taking medication	10 (12.5)	3 (13.0)	7 (12.3)	0.689
	Confusion: Administering the wrong medication due to confusion	4 (5.0)	1 (4.3)	3 (5.3)	1.000
CAREGIVING ERRORS	Forgetting dietary requirements when cooking (e.g., forgetting that the diet was low in salt or low in fats or an allergy to a certain food)	6 (7.5)	2 (8.7)	4 (7.0)	0.623
	Failing to achieve the appropriate texture in the food that had to be given to the person under care	2 (2.5)	1 (4.3)	1 (1.8)	0.427
	Allowing the person cared for to trip and fall during a shower or bath due to not using the proper support or shower chair	5 (6.3)	0 (0.0)	5 (8.8)	0.321
	Injuring your back while making a wrong move during a transfer or movement of the person cared for	9 (11.3)	1 (4.3)	8 (14.0)	0.428
	TOTAL	54 (100)	13 (24.1)	41 (75.9)	

* Fisher’s exact test.

## Data Availability

All data from this study will be openly accessible upon reasonable request.
